# The larval nervous system of the penis worm *Priapulus caudatus* (Ecdysozoa)

**DOI:** 10.1098/rstb.2015.0050

**Published:** 2016-01-05

**Authors:** José M. Martín-Durán, Gabriella H. Wolff, Nicholas J. Strausfeld, Andreas Hejnol

**Affiliations:** 1 Sars International Centre for Marine Molecular Biology, University of Bergen, Thormøhlensgate 55, Bergen 5008, Norway; 2 Department of Neuroscience, School of Mind, Brain, and Behavior, University of Arizona, Tucson, AZ 85721, USA; 3 Center for Insect Science, University of Arizona, Tucson, AZ 85721, USA

**Keywords:** nervous system, Priapulida, arthropoda, nematoda, Ecdysozoa, larva

## Abstract

The origin and extreme diversification of the animal nervous system is a central question in biology. While most of the attention has traditionally been paid to those lineages with highly elaborated nervous systems (e.g. arthropods, vertebrates, annelids), only the study of the vast animal diversity can deliver a comprehensive view of the evolutionary history of this organ system. In this regard, the phylogenetic position and apparently conservative molecular, morphological and embryological features of priapulid worms (Priapulida) place this animal lineage as a key to understanding the evolution of the Ecdysozoa (i.e. arthropods and nematodes). In this study, we characterize the nervous system of the hatching larva and first lorica larva of the priapulid worm *Priapulus caudatus* by immunolabelling against acetylated and tyrosinated tubulin, pCaMKII, serotonin and FMRFamide. Our results show that a circumoral brain and an unpaired ventral nerve with a caudal ganglion characterize the central nervous system of hatching embryos. After the first moult, the larva attains some adult features: a neck ganglion, an introvert plexus, and conspicuous secondary longitudinal neurites. Our study delivers a neuroanatomical framework for future embryological studies in priapulid worms, and helps illuminate the course of nervous system evolution in the Ecdysozoa.

## 1. Introduction

The animal nervous system is the specialized set of cells, tissues and organs responsible for integrating external and internal stimuli and coordinating adequate responses. During evolutionary time, the nervous system has acquired an astonishing level of complexity in bilaterally symmetrical animals (Bilateria), with the appearance of centralized and highly organized neural clusters, such as brains and nerve cords [1]. The presence of centralized nervous systems in distantly related bilaterian groups has raised a vivid debate on the homology (common ancestry) of these structures [2–11], and therefore about the morphological and functional diversification of the nervous system across bilaterian lineages. Insects, and to a minor extent other arthropods, have been key players in almost all these controversies, due to the tripartite organization of their brains and the presence of prominent anterior neuropils called mushroom bodies. These two sophisticated neural features have been homologized with similar anatomical structures in vertebrates and annelids [2,4–7,12], and thus used as argument for the presence of circuit ground patterns that also characterize brains in lineages that have diverged from the last common bilaterian ancestor. However, a proper understanding of the evolution of the arthropod nervous system also requires a detailed morphological, embryological and molecular investigation of often-neglected related bilaterian lineages, in particular those that occupy informative nodes in the phylogeny. Such studies will reveal a better understanding of the evolutionary changes that led to nervous system diversity and how the nervous system architecture relates to the molecular and behavioural repertoire.

Arthropods, onychophorans and tardigrades (Panarthropoda), together with nematodes and nematomorphs (Nematoida), are members of the Ecdysozoa [13]. Recently, molecular phylogenies have placed the priapulid worms (Priapulida), kinorhynchs and loriciferans in a group called Scalidophora, as the sister group taxa to the remaining ecdysozoans (i.e. nematoids and panarthropods) [14–17]. Priapulids, commonly referred to as penis worms, are exclusively marine, mud-dwelling or interstitial animals [18,19]. Despite being among the most abundant fossils in the Early Cambrian [20–22], Priapulida comprise only 19 known extant species [18,19,23]. Adults are sausage-shaped, annulated worms with bodies divided into an anterior retracting introvert with a terminal mouth, and a posterior trunk with a terminal anus and, in some species, a caudal appendage [19]. After external fertilization, priapulid eggs undergo holoblastic radial cleavage, deuterostomic development and formation of a ventral mouth, which are all inferred ancestral characters for the Ecdysozoa [24–27]. Embryonic development results in the formation of a larva, which matures into the adult worm through successive rounds of moulting [28–31]. Morphological and developmental evidence, together with their slow rate of molecular evolution [32] and phylogenetic position support the role of priapulid worms as a key group to understanding the earliest steps of ecdysozoan evolution, and thus deducing ancestral characters to morphologically more diverse ecdysozoan taxa, such as insects.

Studies on the nervous system of the Priapulida are, however, scarce, and mostly focused on adult stages or mature larval stages [30,33–37]. Only recently, immunohistological techniques have been applied to adult specimens of the meiobenthic species *Tubiluchus troglodytes* in order to study the nervous system [38]. In adults and mature priapulid larvae, the central nervous system (CNS) is intraepidermal and comprises a circumoral brain, an unpaired ventral nerve cord and two main ganglia, the neck ganglion at the joint between the introvert and the trunk and the caudal ganglion at the most posterior region of the body [38,39]. Notably, descriptions of a putative priapulid from the Mid-Cambrian, *Ottoia prolifica* [22], identify a paired reflective strand along the ventral midline and have been interpreted as a paired ventral cord [40]. Associated with the CNS, there are nerve plexuses in the pharynx, body wall and caudal appendage, as well as a stomatogastric nerve plexus in the digestive tract [33–35,38,41]. Immunodetection of serotonin and RFamide-like peptides demonstrated the presence of different neural subpopulations in almost all components of the priapulid nervous system [38]. In contrast with our current knowledge of the more mature stages, virtually nothing is known about the embryonic development and early post-embryonic morphology of the nervous system of priapulid worms, which are ultimately essential to understanding the evolution of the great diversity of nervous systems observed in other representatives of the Ecdysozoa.

To gain a better knowledge of the early stages of nervous system formation in priapulid worms, we analysed the immunostaining domains of five antibodies commonly used to characterize neural structures in ecdysozoan animals [2,38,42–51] in hatching larvae and first lorica larvae of the species *Priapulus caudatus* Lamarck, 1816. Immunodetection of acetylated tubulin, tyrosinated tubulin, phosphorylated calcium/calmodulin-dependent protein kinase II (pCaMKII), serotonin and FMRF-like peptides (FLPs) demonstrates that the nervous system of hatching priapulid embryos consists of a circumoral brain, a main ventral nerve, a caudal ganglion and several less conspicuous neurite bundles associated with the buccal scalids, neck and sensory trunk tubuli. The first moulting event implies a significant maturation of the nervous system, with a general increase in the number of neuronal cells and nerve fibres, and the appearance of the neck ganglion. Our study is an important contribution to the study of the Priapulida and improves our understanding of the diversification of the nervous system in the Ecdysozoa, and thus of the evolution of some of the most elaborated neural structures found in animals.

## 2. Material and methods

### (a) Animal collection, fertilization and larva fixation

Adult gravid specimens of *P. caudatus* were collected from Gullmarsfjorden (Fiskebäckskil, Sweden) during the autumn. Dissection of the gonads, fertilization of the oocytes and culture of the embryos were performed as described elsewhere [24]. Embryos hatched 9 days after fertilization, and hatching larvae moulted to the first lorica larvae approximately two weeks thereafter, without any added food source. Before fixation, larvae were relaxed in 0.1% tricaine in filtered seawater (FSW) for 30 s, and immediately fixed in 4% paraformaldehyde (PFA) in FSW for 1 h at room temperature. Fixative was washed out in phosphate buffered saline (PBS) with 0.1% Tween-20 (PTw) before storage in 0.1% sodium azide in PTw at 4°C.

### (b) Immunohistochemistry

Fixed hatching and first lorica larvae were washed three times for 5 min in PTw to remove sodium azide, and perforated afterwards with a thin needle to allow antibody penetration through the larval cuticle. Perforated larvae were transferred into PBS with 0.5% Triton X-100 (PTx) for permeabilization for 2 h at room temperature, and subsequently blocked in 1% bovine serum albumin (BSA) in PTx for 2 h at room temperature. Before adding the primary antibody, larvae were blocked in 10% normal goat serum (NGS) in PTx twice for half an hour. The analysed primary antibodies (mouse anti-acetylated tubulin (Sigma, #T6793), mouse anti-tyrosinated tubulin (Sigma, #T9028), rabbit anti-pCaMKII (Santa Cruz Biotechnology, #sc-12886), rabbit anti-serotonin (Sigma, #S5545) and rabbit anti-FMRFamide (Immunostar, #20091)) were diluted 1 : 100 in 10% NGS in PTx and incubated for approximately 40 h at 4°C. Continuous washes in 1% BSA in PTx for approximately 7 h to remove the primary antibody were followed by blocking in 10% NGS in PTx twice for half an hour and incubation in Alexa-conjugated secondary antibody diluted 1 : 250 in 10% NGS in PTx for approximately 40 h at 4°C. Finally, secondary antibodies were washed out in PTx, and if needed nuclei were counterstained with Sytox Green.

### (c) Imaging

Stained larvae were cleared in Murray's reagent and representative specimens were scanned with a Leica SP5 confocal laser-scanning microscope. Images were analysed in Fiji and Photoshop CS6 (Adobe), and figure plates made with Illustrator CS6 (Adobe). Brightness/contrast and colour balance adjustments were always applied to the whole image, not parts.

## 3. Results

### (a) The early larval nervous system of *Priapulus caudatus*

**Figure 1.**
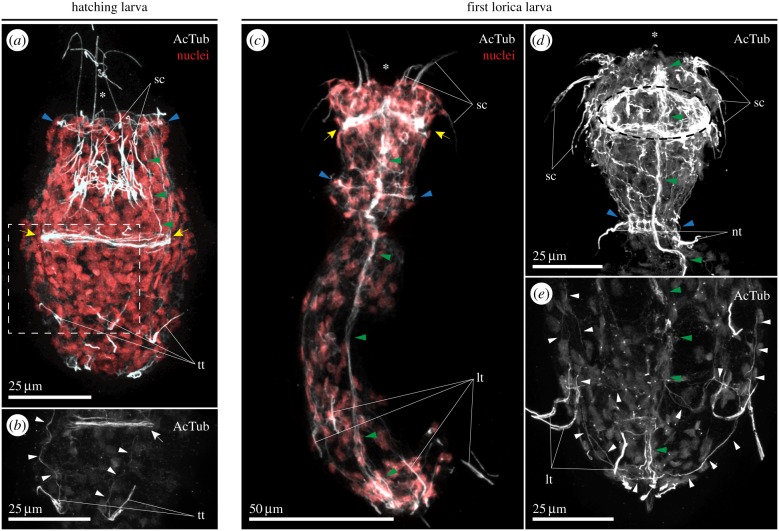
Localization of acetylated tubulin in *P. caudatus* larvae. (*a–e*) Maximal *z*-projections of confocal stacks of whole mount larvae stained against acetylated tubulin (AcTub, in grey) and counterstained with the nuclear marker Sytox Green (red, in *a* and *c*). (*a*) The hatching larva of *P. caudatus* shows a circumoral brain (yellow arrows) and neural commissures at the neck region (neck ganglion; blue arrowheads). The oral scalids and the posterior sensory trunk tubuli are also innervated. (*b*) Detail of the region indicated by a dashed rectangle in (*a*). Thin neural fibres (white arrowheads) project from the sensory trunk tubuli towards the introvert. (*c*) After the first moult, a well-developed ventral nerve cord (green arrowheads) connects the circumoral brain (yellow arrows) with the posterior region of the trunk. The neck ganglion (blue arrowheads) appears more distinct. (*d*) The introvert region of the first lorica larva is rich in neural fibres, with a dense innervation of the scalids from the brain area (black dashed circle; main ventral nerve indicated by green arrowheads and the neck commissures by blue arrowheads). (*e*) Similar to the anterior scalids, the posterior lorica tubuli are strongly innervated, with thin fibres (white arrowheads) projecting from them longitudinally towards the anterior region and posteriorly towards the anal opening, where they meet with the ventral nerve (green arrowheads). In all cases, the asterisk indicates the position of the mouth. (*a,b*) are lateral views, and (*c–e*) are ventral views. lt, lorica tubulus; nt, neck tubulus; sc, scalids; tt, trunk tubulus. Scale bars, 25 µm in (*a,b,d,e*); 50 µm in (*c*).

The hatching larva of *P. caudatus* has a functional anterior introvert with seven primary plus one to three secondary oral scalids (feeding and predatory teeth), a short neck region with a pair of tubuli and a posterior trunk with approximately four trunk tubuli, probably of sensory function [28]. Internally, the hatching larva possesses a well-developed muscular and digestive system [26]. Acetylated tubulin immunoreactivity indicates that the nervous system of hatching larvae consists of a dense circumoral brain and a thin main longitudinal ventral nerve, as well as several less conspicuous nerve fibres (figure 1*a,b*). Among these less evident neurite bundles, there are circular nerve fibres in the neck region and longitudinal neurite bundles that seem to connect the posterior sensory tubuli of the trunk with the introvert neural structures (figure 1*a,b*). The hatching larva of *P. caudatus* is non-feeding and moults into the first lorica larva after approximately two weeks. As the name indicates, this is the first larval stage with a true lorica protecting the trunk. In the first lorica larva, the number of scalids increases, the neck tubuli disappear, and four lorica tubuli are visible in the ventro- and dorso-lateral lorica plates [28]. At this larval stage, the brain and ventral nerve appear more developed and seem to include a greater number of nerve fibres (figure 1*c,d*). The neck commissures are now packed into a well-formed ganglion, and many secondary nerve fibres connect this structure with the circumoral brain (figure 1*c,d*), defining the introvert nerve plexus. The lorica tubuli project longitudinal nerve fibres that connect posteriorly in the anal region and run anteriorly towards the main introvert neural structures (figure 1*e*). Additionally, transverse commissures appear to connect these secondary neurite bundles between them and with the main ventral nerve (figure 1*e*).

**Figure 2.**
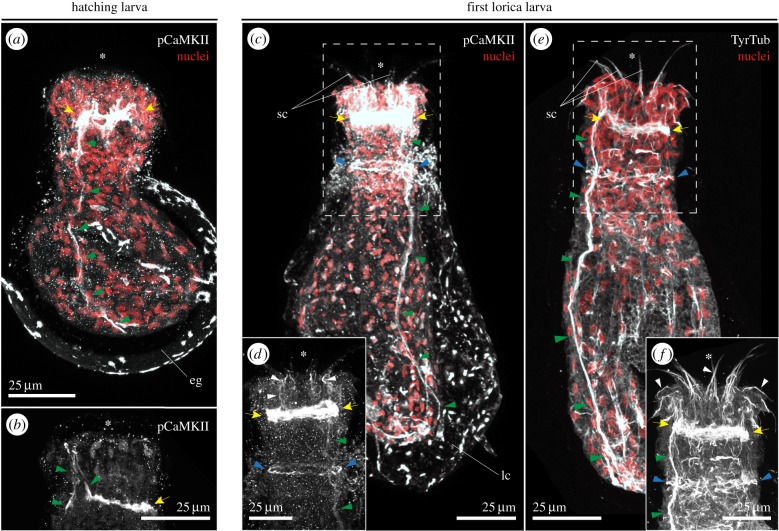
Localization of pCaMKII and tyrosinated tubulin in *P. caudatus* larvae. (*a–f*) Maximal *z*-projections of confocal stacks of whole mount larvae stained against pCaMKII (in grey) or tyrosinated tubulin (TyrTub, in grey) and counterstained with the nuclear marker Sytox Green (red, in *a*, *c* and *e*). (*a*) In the hatching larva of *P. caudatus*, antisera against phosphorylated CaMKII localize in the circumoral brain nerves (yellow arrows) and ventral nerve (green arrowheads). (*b*) The ventral nerve (green arrowheads) connects to the brain (yellow arrow) anteriorly, folding inside the body as the introvert retracts. (*c,d*) In the first lorica larva, immunoreactivity to pCaMKII is stronger in the brain (yellow arrows), the neck ganglion (blue arrowheads), ventral nerve (green arrowheads), and nerve projections towards the buccal scalids (white arrowheads in *d*). (*e,f*) Immunoreactivity against TyrTub appears in the first lorica larva, greatly concentrated in the circumoral brain (yellow arrows), neck ganglion (blue arrowheads), main ventral nerve (green arrowheads) and the innervation of the scalids (white arrowheads in *f*). In all cases, the asterisk indicates the position of the mouth. The staining in the eggshell and the lorica in (*a*) and (*c*) is background. All panels are ventral–lateral views. eg, eggshell; lc, lorica; sc, scalids. Scale bars, 25 µm in all panels.

pCaMKII immunoreactivity is restricted to the brain and the main ventral nerve of both the hatching larva and the first lorica larva (figure 2*a–d*). While pCaMKII labelling is not observed in the neck region in the hatching larva of *P. caudatus* (figure 2*b*), immunoreactivity in the neck ganglion is evident with the first moult (figure 2*d*), together with the labelling of fine nerve fibres projecting from the anterior region of the neuropil towards the buccal scalids (figure 2*d*). Finally, tyrosinated tubulin immunoreactivity was not consistently observed in hatching larvae, and only reliably detected in the first lorica larvae. At this stage, tyrosinated tubulin antibody labelled the brain, neck ganglion and ventral nerve (figure 2*e,f*). Altogether, the immunolabelling of acetylated and tyrosinated tubulin and pCaMKII show that the CNS of priapulid embryos at hatching is already composed of a circumoral brain and a main ventral nerve ending in a caudal ganglion. Additionally, neurite bundles associated with the sensory trunk tubuli and scalids make up the peripheral nervous system (PNS). With the first moulting event, the nervous system experiences a significant increase in complexity, with a general rise in the number of neurite fibres in both the CNS and the PNS.

### (b) The serotonergic nervous system

**Figure 3.**
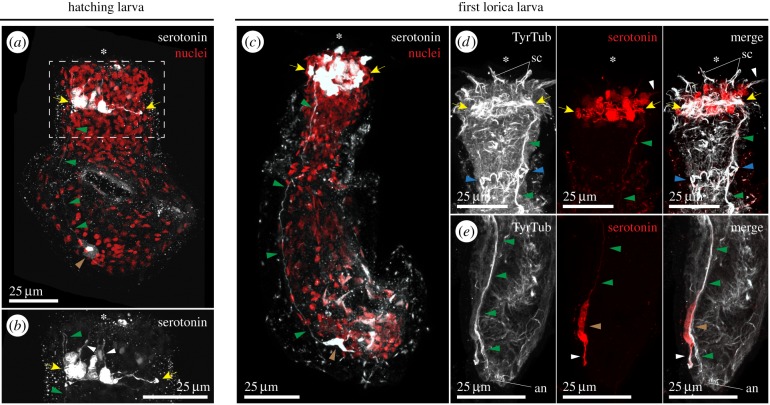
The larval serotonergic nervous system of *P. caudatus*. (*a–e*) Maximal *z*-projections of confocal stacks of whole mount larvae stained with antisera against serotonin (*a–c*, in grey; *d* and *e*, in red) or tyrosinated tubulin (TyrTub; *d* and *e*, in grey) and counterstained with the nuclear marker Sytox Green (red, in *a*,*c*). (*a*) The serotonergic nervous system of the hatching larva comprises perikarya around the circumoral brain (yellow arrows), an axonal tract in the ventral nerve (green arrowheads) and one cell at the caudal ganglion (brown arrowhead). (*b*) Magnification of the squared region in (*a*). Serotonergic cells in the brain are bipolar, with one axon projecting towards the anterior end (white arrowheads) and the other one projecting towards the neuropil (yellow arrows; ventral nerve indicated by green arrowheads). (*c*) In the first lorica larva, the number of serotonin-positive cells in the brain increases (yellow arrows), the ventral nerve (green arrowheads) is more conspicuous and one serotonin-positive cell is still observed in the caudal ganglion (brown arrowhead). (*d*) The serotonin-positive cells are located anterior to the neuropil (yellow arrows, as observed with TyrTub; the blue arrowheads indicate the neck ganglion, and the green arrowheads the ventral nerve), with the anterior cells projecting one axon towards the scalids (white arrowhead). (*e*) In the posterior region, the serotonergic cell of the caudal ganglion (brown arrowhead) projects its posterior axon (white arrowhead) outside the ventral nerve (green arrowheads). In all cases, the asterisk indicates the position of the mouth. All panels are ventral–lateral views. an, anus; sc, scalids. Scale bars, 25 µm in all panels.

Serotonin-positive cells localize to the circumoral brain and caudal ganglion of the hatching larva of *P. caudatus* (figure 3*a,b*). In the brain, serotonin-positive cells are bipolar, projecting one axon towards the anterior end of the introvert, where the scalids are located, and the other axon towards the neuropil (figure 3*b*). One single bipolar serotonin-positive cell is observed in the caudal ganglion, which projects one axon posteriorly towards the anus and another one anteriorly towards the brain through the main ventral nerve (figure 3*a*). With our data, we cannot discriminate whether the serotonin-positive axon of the ventral neurites extends from the circumoral brain or from the caudal ganglion. After the first moult, the number of serotonin-positive cells increases, although the overall distribution remains (figure 3*c*). In the brain region, serotonergic cells innervate the scalids and distribute anteriorly of the neuropil (figure 3*d*). In the posterior trunk, the caudal ganglion contains one bipolar serotonin-positive cell, which projects the posterior axon outside the main ventral nerve (figure 3*e*). The serotonergic nervous system of the first larval stages of *P. caudatus* is thus restricted to the main elements of the CNS, in contrast with the situation observed in adult priapulids, where serotonin-positive cells are widespread also in the PNS [38].

### (c) FMRFamide-like peptides in *Priapulus caudatus* early larval stages

**Figure 4.**
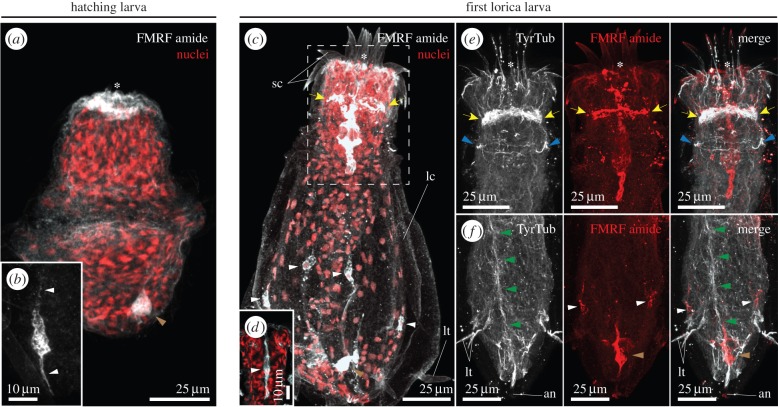
Localization of FLPs in *P. caudatus* larvae. (*a–f*) Maximal *z*-projections of confocal stacks of whole mount larvae stained with an anti-FMRFamide antibody (*a–d*, in grey; *d* and *e*, in red) or against tyrosinated tubulin (TyrTub; *e* and *f*, in grey) and counterstained with the nuclear marker Sytox Green (red, in *a*, *c* and *d*). (*a*) In the hatching larva, FLPs are detected in a bipolar cell (white arrowheads in magnification in (*b*) at the caudal ganglion (brown arrowhead). (*c*) After the first moult, FLP immunoreactivity is observed in the neuropil (yellow arrows), caudal ganglion (brown arrowhead) and several cells of the trunk (white arrowheads). (*d*) The epithelial cells lining the lumen of the buccal cavity exhibit immunoreactivity for FLPs (white arrowhead). (*e*) Magnification of the squared region in (*c*). FLP immunoreactivity in the introvert is observed in the brain neuropil (yellow arrows; neck ganglion indicated by blue arrowheads). (*f*) In the posterior region, FLPs are observed in two cells of the caudal ganglion (brown arroheads) at the end of the main ventral nerve (green arrowheads) and in cells of the thin neurite bundles departing from the trunk tubuli (white arrowheads). In all cases, the asterisk indicates the position of the mouth. All panels are ventral–lateral views. lc, lorica; lt, lorica tubulus; sc, scalids. Scale bars, 25 µm in (*a,c,e,f*); 10 µm in (*b,d*).

The hatching larva of *P. caudatus* exhibits immunoreactivity to FLPs in one cell at the posterior region of the trunk (figure 4*a*), presumably in one bipolar cell of the caudal ganglion (figure 4*b*). Immunoreactivity at the buccal opening is consistent among hatching larvae (figure 4*a*), but does not seem to be associated with any particular cells, and thus it might be unspecific binding of the antibody. The moulting of the hatching larva into the first lorica larva significantly affects the distribution of FLPs in the nervous system. The circumoral brain of the first lorica larva appears immunoreactive for FLPs, as well as the caudal ganglion and several isolated cells along the trunk (figure 4*c*). In the introvert, the FLP-positive region localizes to the neuropil, as well as in cells of the inner epithelium of the buccal cavity (figure 4*e*). This staining was present in all analysed larvae, and it seems to affect the luminal cells (figure 4*d*). Posteriorly, FLP-positive cells of the trunk appear associated with the lorica tubuli and the neurite bundles that depart from them towards the anterior CNS (figure 4*f*). Therefore, FLPs appear to localize in both the CNS and the PNS of the first lorica larvae of *P. caudatus*.

## 4. Discussion

### (a) The early larval nervous system of *Priapulus caudatus*

**Figure 5.**
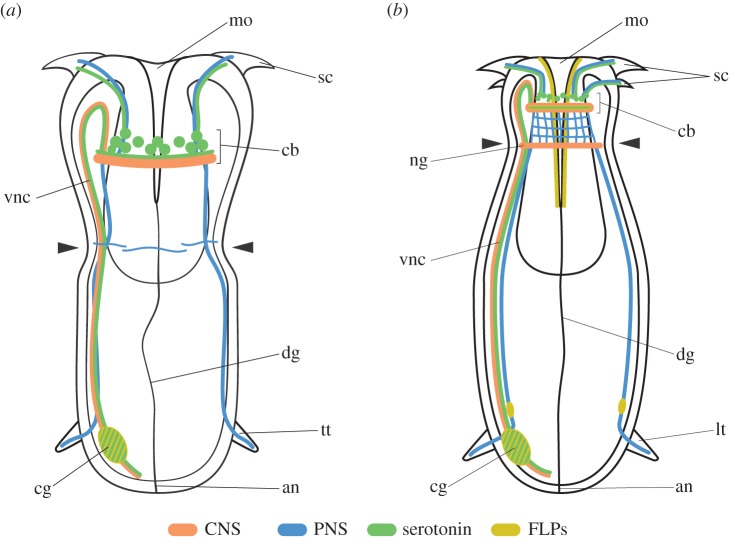
The early larval nervous system of *P. caudatus*. (*a,b*) Schematic of the nervous system of the hatching larva and first lorica larva of *P. caudatus*. (*a*) In the hatching larva, the CNS (in orange) consists of a circumoral brain, an unpaired ventral nerve and a caudal ganglion (serotonin- and FLP-positive). In the brain, all serotonin cell bodies localize anterior to the neuropil. From the brain, neurite bundles innervate the scalids. The PNS (in blue) includes neck commissures and nerves connecting the trunk tubuli with the brain. (*b*) In the first lorica larva, the nervous system is more mature. The CNS consists of the circumoral brain, the ventral nerve, and a neck and a caudal ganglion. The brain neuropil is also FLP-positive. The PNS includes secondary nerves innervating the scalids, connecting the brain and the neck ganglion, and innervating the lorica tubuli (also FLP-positive). The epithelium of the buccal cavity is immunoreactive to FLPs. Drawings are not to scale and are lateral views, with the anterior to the top and ventral to the left. an, anus; cb, circumoral brain; cg, caudal ganglion; dg, digestive tract; lt, lorica tubule; mo, mouth; ng, neck ganglion; sc, scalid; tt, trunk tubule; vnc, ventral nerve cord.

Studies on the nervous system of priapulid worms are scarce, and so far exclusively focused on adult stages and larval forms obtained from direct field sampling which already have a lorica, and thus correspond to late larval stages [23,30,33–38,41]. In our study, we analysed the immunoreactivity patterns of five antibodies routinely used in immunohistochemical neuroanatomy (figures 1–4) to characterize the earliest post-embryonic stages of nervous system formation in larval forms obtained by *in vitro* fertilization. Our results show that the CNS of the hatching larva consists of a circumoral brain, an apparently unpaired longitudinal ventral nerve, and a caudal ganglion (figure 5*a*). The circumoral brain has a bipartite organization, with the somata (at least the serotonin-positive cells) located anteriorly to the neuropil (figure 3*b*) [52]. This type of organization seems to be common also in the Eupriapulida [33,41], but differs from the situation observed in the Tubiluchidae, where the brain includes serotonergic somata located both anteriorly and posteriorly to the central neuropil [34,35,38]. The ventral longitudinal nerve is unpaired and leaves the circumoral brain anteriorly, turning backwards towards the posterior anus at the anterior most region of the introvert. In the hatching larva, the main ventral neurite bundle is thin, probably formed by a very limited number of axonal tracts, and serotonin-positive (figures 1–3). We did not observe any nuclei associated with the main ventral longitudinal nerve. At its posterior end, there is a serotonin- and FLP-positive caudal ganglion. Additionally, the hatching larva presents thin neck commissures, and peripheral innervation of the buccal scalids and of the trunk tubuli (figures 1, 3 and 5*a*). The presence of axonal tracts leaving the trunk tubuli suggests that these structures are sensory organs of the larva [38], although alternative and/or complementary roles (e.g. adhesion) have been proposed [53]. Altogether, the nervous system found in hatching larvae indicates that the embryos of *P. caudatus* leave the eggshell with a basic layout of the adult priapulid nervous system. To date, the only neural gene expression reported in priapulid embryos is that of *orthodenticle* (*otx*) [24]. *otx* is expressed in the ventral ectoderm of the introvert and in a ring around the introvert–trunk boundary at the introvertula stage. According to the results shown in this study, this expression would correspond to the circumoral brain of the hatching larva, once the introvert has retracted inside the trunk during late embryogenesis [26]. Our study thus offers the neuroanatomical framework for future embryological studies on the development of the nervous system of *P. caudatus*.

The first moulting event leads to a significant change in the complexity of the nervous system of *P. caudatus* (figure 5*b*), as has been also described for other organ systems such as the digestive tract, the musculature and the external morphology of the cuticle [26,28]. The CNS, as revealed by the immunoreactivity pattern for pCaMKII and tyrosinated tubulin (figure 2), includes a well-developed neck ganglion, and thus appears similar to the organization observed in adult priapulids [19,33,38,39]. The other components, namely the circumoral brain, the main ventral nerve and the caudal ganglion, contain more somata and nerve fibres. Important changes are observed in the PNS, where many neurite bundles connecting the brain with the neck ganglion are observed. In addition, the connection between the lorica tubuli and the CNS increases in complexity, by including FLP-positive cells along the neural tracts. Despite this significant change in the organization of the nervous system of the first lorica larva of *P. caudatus*, important features observed in the nervous system of adult priapulids are still missing. We did not find any evidence of serotonin signal around the gut or in the body wall nerve plexus, as observed for instance in *T. troglodytes* [38], and the pharyngeal/introvert plexus is also significantly more simple than that observed in adult stages [38,54]. The distribution of the FLPs is also more localized than in adult priapulids [38]. In addition, the adult *T. troglodytes* has an orthogonal pattern of neurites [38], which seems to be absent in at least these early larval stages of *P. caudatus*. Therefore, the basic anatomical organization of the priapulid nervous system is attained at the first lorica larva stage, although subsequent rounds of moulting must relate to the appearance of the mature features of the nervous system of adult stages, probably associated with the onset of predatory behaviours.

### (b) Implications for the evolution of the nervous system in the Ecdysozoa

Evolutionary discussions on the diversification of the nervous system within the Ecdysozoa are hampered by the limited availability of neuroanatomical data regarding the Priapulida, and Scalidophora generally. Moreover, the scarce studies on priapulid worms are entirely restricted to adult stages and late larval forms, with almost nothing known regarding the embryonic formation of the nervous system. The situation is even more severe for the other two scalidophoran lineages, namely the Kinorhyncha and the Loricifera, for which general data on their embryogenesis are absent or extremely limited [55,56]. Therefore, our characterization of the nervous system of the hatching larva and first lorica larva of *P. caudatus* is an important first step towards closing this gap of knowledge.

**Figure 6.**
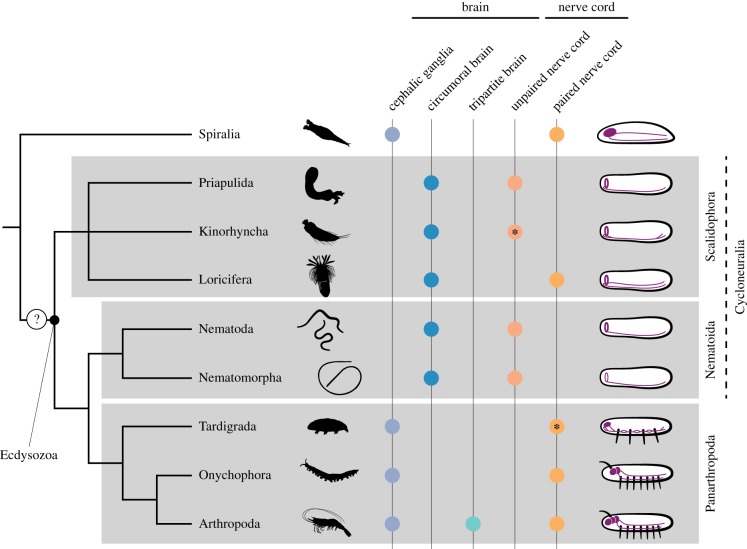
The diversity of central nervous systems in the Ecdysozoa. Distribution of major anatomical characteristics of the CNS in the different ecdysozoan lineages, compared with the closest relative outgroup (the Spiralia). Ecdysozoan phylogeny according to Pisani *et al.* [17]. Altogether, the distribution of the plotted characters favours considering an unpaired ventral nerve cord as the ancestral architecture of the ecdysozoan nervous system, although the nature of the brain is still unclear. The asterisk in the Kinorhyncha indicates that the ventral nerve cord is unpaired in the trunk and bifurcates anteriorly and posteriorly, as shown in the schematic on the right. The asterisk in the Tardigrada indicates that the ventral cord ganglia are unpaired, but the neurite connections between them are paired, as shown in the schematic on the right. The dashed line uniting the Cycloneuralia indicates that there are contrasting data supporting this grouping (see main text for references). Drawings are not to scale.

Inferring the ancestral form of the scalidophoran, and ecdysozoan, nervous system is thus a hard task, as it becomes obstructed by the problematic logistics of comparing late embryonic/early larval data (this study and taxa from the Nematoida and Panarthropoda) with the anatomy of more mature stages (other members of the Priapulida, the Kinorhyncha and the Loricifera). Nevertheless, general evolutionary hypotheses can be formulated, which can ultimately serve as matters for further study. If, for the sake of simplicity, we focus on the CNS, the earliest and simplest anatomical form comprises a circumoral brain and an unpaired ventral nerve in *P. caudatus* at least in the larva. However, palaeontological evidence suggests that the adult forms of the Mid-Cambrian priapulid *O. prolifica* [22] possessed a paired ventral cord [39]. The basic organization found in priapulid larvae is also observed in kinorhynchs and loriciferans (figure 6), although the ventral nerve cord bifurcates anteriorly to connect with the brain and also posteriorly after the caudal ganglion in the Kinoryncha [57,58], and is paired in the Loricifera [59]. In nematodes and nematomorphs there is a main unpaired ventral nerve cord [60,61], whereas in panarthropods the ventral nerve cord is paired (in the Tardigrada the nerve cord ganglia are unpaired) [62,63] (figure 6). In the Spiralia (e.g. Gnathifera, Trochozoa) the main neural tracts found in the ventro-lateral body region are paired, although in several annelids a median nerve is also present [64–68] (see also Hejnol and Lowe [69]) and renders the reconstruction of a paired versus unpaired nerve cord ambiguous. Principally, the distribution of an unpaired ventral nerve cord within Scalidophora and Nematoida favours a reduction event at the base of the Ecdysozoa and thus the secondary separation of the major ventral nerve into two main ventral tracts in loriciferans and panarthropods (figure 6). However, in the nematode *Pontonema vulgare* [61] less prominent, paired, ventro-lateral nerves are present in addition to the ventral nerve cord and could hint to the presence of a median and two lateral cords as the ancestral condition which in the course of evolution got elaborated and/or reduced in the different lineages. In this regard, the comparative study of the mediolateral patterning system [7,70] between those lineages with unpaired ventral nerve cords and those with paired ventral nerve cords might shed light into the homology of the nerve tracts and help to reconstruct possible developmental events responsible for the evolution of this trait.

An important and highly debated issue is the nature of the brain in the last common ancestor of the Ecdysozoa [3–7,10,11]. Priapulids, kinorhynchs, loriciferans, nematomorphs and nematodes have a circumoral brain composed of a ring neuropil with anterior and posterior somata, which contrasts with the circumoral commissures found in other ecdysozoans [52] (figure 6). This trait was used to unite all these lineages into the Cycloneuralia [14,71], although most recent molecular phylogenies recover this grouping as paraphyletic [15–17]. On the other hand, the Panarthropoda shows more or less developed anterior neural concentrations [43,44,50,63,72–74] (figure 6), but the homology of these structures between tardigrades, onychophorans and arthropods is still debated [43,44,50,72]. Within Ecdysozoa, it is in arthropods that the brain attains the highest level of sophistication [63,75], probably related to the increase in the number of cephalic segments and the development of more specialized head structures. In this lineage, the brain is considered to be composed of three main neuromeres (protocerebrum, deuterocerebrum and tritocerebrum), and thus has been referred to as a tripartite brain [76,77]. Frustratingly, similar terminology has been used to describe the circumoral brain of some priapulids [38], based on the presence of three histological layers (anterior somata, central neuropil, posterior somata). This situation is not observed in the larva and adult of *P. caudatus* (figure 3), where histological methods only reveal the anterior somata and the neuropil, and thus a bipartite brain (see also discussion above). However, the use of these terms to describe the priapulid brain can be misleading, as the ‘tripartite’ anatomical organization should refer to the segmental nature of the brain and should include the linearity of ‘segmentation’ genes that are required for such segments to develop. Only if orthologous genes were to be expressed in relation to each of the histological layers of the priapulid brain would there be grounds for applying the same terminology to priapulids. Regardless of the terminology used, the distribution of brain architectures in the different lineages of the Ecdysozoa and outgroup taxa suggests two alternative scenarios for the evolution of this neuroanatomical component (figure 6). On the one hand, the distribution of a circumoral brain among the Ecdysozoa might indicate that this was the most probable brain architecture in the last common ecdysozoan ancestor. On the other hand, the presence of brain ganglia in the Panarthropoda and in taxa outside the Ecdysozoa might indicate that the circumoral brain evolved secondarily and independently in members of the Scalidophora and Nematoida. Further embryological, molecular and physiological data are thus required to fully understand the neuroanatomy of the brain of priapulids, kinorhynchs, loriciferans, nematodes and nematomorphs, and ultimately attain a more accurate picture of the course of nervous system evolution in the Ecdysozoa.

## 5. Conclusion

In this study, we characterize the earliest post-embryonic stages of nervous system development in the priapulid worm *P. caudatus*. The immunoreactivity patterns of five different antibodies commonly used in neuroanatomical analyses demonstrate that priapulid embryos hatch with a nervous system composed of a circumoral brain and an apparently unpaired ventral nerve ending in a caudal ganglion. Additionally, thin neurite bundles innervate the sensory organs of the larva, namely the buccal scalids and the trunk tubuli. The first moulting event in the life cycle of *P. caudatus* implies a significant maturation of the nervous system, which acquires features already seen in adult priapulids, namely the presence of a neck ganglion, a well-developed introvert plexus, and more conspicuous secondary longitudinal nerve tracts. Our results are in agreement with previous morphological observations in adult stages of *P. caudatus* and other priapulid worms [33,38], and deliver the adequate neuroanatomical framework for future embryological studies on *P. caudatus*. In the light of our current knowledge of the ecdysozoan phylogenetic relationships, our results support considering that the ancestral nervous system of the Ecdysozoa might have comprised an unpaired ventral nerve cord, but the architecture of the brain is still unclear. Therefore, further work will be necessary to better understand the exact evolutionary and anatomical relationships between *a priori* simpler brains, such as those found in priapulid worms, and those more elaborated central nervous systems observed in arthropods.

## References

[1] Schmidt-Rhaesa A 2007 The evolution of organ systems. Oxford, UK: Oxford University Press.

[2] Wolff GH, Strausfeld NJ 2015 Genealogical correspondence of mushroom bodies across invertebrate phyla. Curr. Biol. 25, 38–44. (10.1016/j.cub.2014.10.049) 25532890

[3] Pani AM, Mullarkey EE, Aronowicz J, Assimacopoulos S, Grove EA, Lowe CJ 2012 Ancient deuterostome origins of vertebrate brain signalling centres. Nature 483, 289–294. (10.1038/nature10838) 22422262 PMC3719855

[4] Hirth F 2010 On the origin and evolution of the tripartite brain. Brain Behav. Evol. 76, 3–10. (10.1159/000320218) 20926853

[5] Strausfeld NJ, Hirth F 2013 Deep homology of arthropod central complex and vertebrate basal ganglia. Science 340, 157–161. (10.1126/science.1231828) 23580521

[6] Hirth F, Kammermeier L, Frei E, Walldorf U, Noll M, Reichert H 2003 An urbilaterian origin of the tripartite brain: developmental genetic insights from *Drosophila*. Development 130, 2365–2373. (10.1242/dev.00438) 12702651

[7] Arendt D, Denes AS, Jékely G, Tessmar-Raible K 2008 The evolution of nervous system centralization. Phil. Trans. R. Soc. B 363, 1523–1528. (10.1098/rstb.2007.2242) 18192182 PMC2614231

[8] Arendt D, Nübler-Jung K 1997 Dorsal or ventral: similarities in fate maps and gastrulation patterns in annelids, arthropods and chordates. Mech. Dev. 61, 7–21. (10.1016/S0925-4773(96)00620-X) 9076674

[9] Gerhart J, Lowe CJ, Kirschner M 2005 Hemichordates and the origin of chordates. Curr. Opin. Genet. Dev. 15, 461–467. (10.1016/j.gde.2005.06.004) 15964754

[10] Holland LZ, Carvalho JE, Escriva H, Laudet V, Schubert M, Shimeld SM, Yu JK 2013 Evolution of bilaterian central nervous systems: a single origin? EvoDevo 4, 27 (10.1186/2041-9139-4-27) 24098981 PMC3856589

[11] Holland ND 2003 Early central nervous system evolution: an era of skin brains? Nat. Rev. Neurosci. 4, 617–627. (10.1038/nrn1175) 12894237

[12] Steinmetz PRH et al. 2010 *Six3* demarcates the anterior-most developing brain region in bilaterian animals. EvoDevo 1, 14 (10.1186/2041-9139-1-14) 21190549 PMC3025827

[13] Dunn CW, Giribet G, Edgecombe GD, Hejnol A 2014 Animal phylogeny and its evolutionary implications. Annu. Rev. Ecol. Evol. Syst. 45, 371–395. (10.1146/annurev-ecolsys-120213-091627)

[14] Dunn CW et al. 2008 Broad phylogenomic sampling improves resolution of the animal tree of life. Nature 452, 745–749. (10.1038/nature06614) 18322464

[15] Hejnol A et al. 2009 Assessing the root of bilaterian animals with scalable phylogenomic methods. Proc. R. Soc. B 276, 4261–4270. (10.1098/rspb.2009.0896) PMC2817096 19759036

[16] Borner J, Rehm P, Schill RO, Ebersberger I, Burmester T 2014 A transcriptome approach to ecdysozoan phylogeny. Mol. Phylogenet. Evol. 80, 79–87. (10.1016/j.ympev.2014.08.001) 25124096

[17] Pisani D, Carton R, Campbell LI, Akanni WA, Mulville E, Rota-Stabelli O 2013 An overview of arthropod genomics, mitogenomics, and the evolutionary origins of the arthropod proteome. In Arthropod biology and evolution (eds Minelli A, Boxshall G, Fusco G), pp. 41–61. Berlin, Germany: Springer.

[18] Nielsen C 2012 Animal evolution. Oxford, UK: Oxford University Press.

[19] Schmidt-Rhaesa A 2013 Priapulida. In Handbook of zoology—Nematomorpha, Priapulida, Kinorhyncha, Loricifera (ed. Schmidt-Rhaesa A), pp. 147–180. Berlin, Germany: Walter e Gruyter GmbH.

[20] Huang DY, Vannier J, Chen JY 2004 Recent Priapulidae and their Early Cambrian ancestors: comparisons and evolutionary significance. Geobios 37, 217–228. (10.1016/j.geobios.2003.04.004)

[21] Vannier J, Calandra I, Gaillard C, Żylińska A 2010 Priapulid worms: pioneer horizontal burrowers at the Precambrian–Cambrian boundary. Geology 38, 711–714. (10.1130/G30829.1)

[22] Smith MR, Harvey THP, Butterfield NJ 2015 The macro- and microfossil record of the Cambrian priapulid *Ottoia*. Palaeontology 58, 705–721. (10.1111/pala.12168)

[23] Lemburg C 1999 Ultrastrukturelle Untersuchungen an den Larven von *Halicryptus spinulosus* und *Priapulus caudatus*. Hypothesen zur Phylogenie der Priapulida und deren Bedeutung fur die Evolution der Nemathelminthes. Göttingen, Germany: Universität Göttingen.

[24] Martín-Durán JM, Janssen R, Wennberg S, Budd GE, Hejnol A 2012 Deuterostomic development in the protostome *Priapulus caudatus*. Curr. Biol. 22, 2161–2166. (10.1016/j.cub.2012.09.037) 23103190

[25] Wennberg SA, Janssen R, Budd GE 2008 Early embryonic development of the priapulid worm *Priapulus caudatus*. Evol. Dev. 10, 326–338. (10.1111/j.1525-142X.2008.00241.x) 18460094

[26] Martín-Durán JM, Hejnol A 2015 The study of *Priapulus caudatus* reveals conserved molecular patterning underlying different gut morphogenesis in the Ecdysozoa. BMC Biol. 13, 29 (10.1186/s12915-015-0139-z) 25895830 PMC4434581

[27] Zhinkin L 1949 Early stages in the development of *Priapulus caudatus*. Dok. Akad. Nauk. 65, 409–412.

[28] Wennberg S, Janssen R, Budd GE 2009 Hatching and earliest larval stages of the priapulid worm *Priapulus caudatus*. Invertebr. Biol. 128, 157–171. (10.1111/j.1744-7410.2008.00162.x)

[29] Janssen R, Wennberg SA, Budd GE 2009 The hatching larva of the priapulid worm *Halicryptus spinulosus*. Front. Zool. 6, 8 (10.1186/1742-9994-6-8) 19470151 PMC2693540

[30] Higgins RP, Storch V, Shirley TC 1993 Scanning and transmission electron microscopical observations on the larvae of *Priapulus caudatus* (Priapulida). Acta Zool. 74, 301–319. (10.1111/j.1463-6395.1993.tb01245.x)

[31] Lang K 1953 On the morphology of the larva of *Priapulus caudatus* Lam. Arkiv. Zool. 41, 1–9.

[32] Webster BL, Copley RR, Jenner RA, Mackenzie-Dodds JA, Bourlat SJ, Rota-Stabelli O, Littlewood DTJ, Telford MJ 2006 Mitogenomics and phylogenomics reveal priapulid worms as extant models of the ancestral Ecdysozoan. Evol. Dev. 8, 502–510. (10.1111/j.1525-142X.2006.00123.x) 17073934

[33] Scharff R 1885 On the skin and nervous system of *Priapulus* and *Halicryptus*. Q. J. Microsc. Sci. 25, 193–213.

[34] Calloway CB 1975 Morphology of the introvert and associated structures of the priapulid *Tubiluchus corallicola* from Bermuda. Mar. Biol. 31, 161–174. (10.1007/BF00391628)

[35] Rehkämper G, Storch V, Alberti G, Welsch U 1989 On the fine structure of the nervous system of *Tubiluchus philippinensis* (Tubiluchidae, Priapulida). Acta Zool. 70, 111–120. (10.1111/j.1463-6395.1989.tb01060.x)

[36] Storch V, Higgins RP 1991 Scanning and transmission electron microscopic observations on the larva of *Halicrypus spinulosus* (Priapulida). J. Morph. 210, 175–194. (10.1002/jmor.1052100207) 29865560

[37] Lemburg C 1995 Ultrastructure of the introvert and associated structures of the larvae of *Halicryptus spinulosus* (Priapulida). Zoomorphology 115, 11–29. (10.1007/BF00397931)

[38] Rothe BH, Schmidt-Rhaesa A 2010 Structure of the nervous system in *Tubiluchus troglodytes* (Priapulida). Invertebr. Biol. 129, 39–58. (10.1111/j.1744-7410.2010.00185.x)

[39] Storch V 1991 Priapulida. In Microscopic anatomy of invertebrates (eds Harrison FW, Ruppert EE), pp. 333–350. New York, NY: Wiley-Liss.

[40] Conway Morris S 1977 Fossil priapulid worms. Spec. Papers Palaeontol. 20, 1–155.

[41] Joffe BI, Kotikova EA 1988 Nervous system of *Priapulus caudatus* and *Halicryptus spinulosus*. Proc. Zool. Inst. USSR Acad. Sci. 183, 52–77.

[42] Mayer G, Whitington PM 2009 Neural development in Onychophora (velvet worms) suggests a step-wise evolution of segmentation in the nervous system of Panarthropoda. Dev. Biol. 335, 263–275. (10.1016/j.ydbio.2009.08.011) 19683520

[43] Mayer G, Whitington PM, Sunnucks P, Pflüger HJ 2010 A revision of brain composition in Onychophora (velvet worms) suggests that the tritocerebrum evolved in arthropods. BMC Evol. Biol. 10, 255 (10.1186/1471-2148-10-255) 20727203 PMC2933641

[44] Persson DK, Halberg KA, Jørgensen A, Møbjerg N, Kristensen RM 2012 Neuroanatomy of *Halobiotus crispae* (Eutardigrada: Hypsibiidae): Tardigrade brain structure supports the clade Panarthropoda. J. Morphol. 273, 1227–1245. (10.1002/jmor.20054) 22806919

[45] Gross V, Mayer G 2015 Neural development in the tardigrade *Hypsibius dujardini* based on anti-acetylated *α*-tubulin immunolabeling. EvoDevo 6, 12 (10.1186/s13227-015-0008-4) 26052416 PMC4458024

[46] Siddiqui SS, Aamodt E, Rastinejad F, Culotti J 1989 Anti-tubulin monoclonal antibodies that bind to specific neurons in *Caenorhabditis elegans*. J. Neurosci. 9, 2963–2972. 2475594 10.1523/JNEUROSCI.09-08-02963.1989 PMC6569711

[47] Zantke J, Wolff C, Scholtz G 2008 Three-dimensional reconstruction of the central nervous system of *Macrobiotus hufelandi* (Eutardigrada, Parachela): implications for the phylogenetic position of Tardigrada. Zoomophology 127, 21–36. (10.1007/s00435-007-0045-1)

[48] Harzsch S, Anger K, Dawirs RR 1997 Immunocytochemical detection of acetylated alpha-tubulin and *Drosophila* synapsin in the embryonic crustacean nervous system. Int. J. Dev. Biol. 41, 477–484. 9240564

[49] Maxmen A, Browne WE, Martindale MQ, Giribet G 2005 Neuroanatomy of sea spiders implies an appendicular origin of the protocerebral segment. Nature 437, 1144–1148. (10.1038/nature03984) 16237442

[50] Eriksson BJ, Budd GE 2000 Onychophoran cephalic nerves and their bearing on our understanding of head segmentation and stem-group evolution of Arthropoda. Arthropod. Struct. Dev. 29, 197–209. (10.1016/S1467-8039(00)00027-X) 18088927

[51] Brenneis G, Ungerer P, Scholtz G 2008 The chelifores of sea spiders (Arthropoda, Pycnogonida) are the appendages of the deutocerebral segment. Evol. Dev. 10, 717–724. (10.1111/j.1525-142X.2008.00285.x) 19021742

[52] Schmidt-Rhaesa A, Rothe BH 2014 Brains in Gastrotricha and Cycloneuralia—a comparison. In Deep metazoan phylogeny: The backbone of the tree of life. New insights from analyses of molecules, morphology, and theory of data analysis (eds Wägele JW, Bartolomaeus T), pp. 93–104. Berlin, Germany: De Gruyter.

[53] Storch V, Alberti G 1985 Ultrastructural investigation of the integument of *Tubiluchus philippinensis* (Priapulida, Tubiluchidae). Zool. Scr. 14, 265–272. (10.1111/j.1463-6409.1985.tb00196.x)

[54] Apel W 1885 Beitrag zur Anatomie und Histologie des *Priapulus caudatus* (Lam) und des *Halicryptus spinulosus* (v. Siebold). Z. Wiss. Zool. 42, 459–529.

[55] Kozloff EN 2007 Stages of development, from first cleavage to hatching, of an *Echinoderes* (Phylum Kinorhyncha: Class Cyclorhagida). Cah. Biol. Mar. 48, 199–206.

[56] Kristensen RM 2002 An introduction to Loricifera, Cycliophora, and Micrognathozoa. Integr. Comp. Biol. 42, 641–651. (10.1093/icb/42.3.641) 21708760

[57] Herranz M, Pardos F, Boyle MJ 2013 Comparative morphology of serotonergic-like immunoreactive elements in the central nervous system of kinorhynchs (Kinorhyncha, Cyclorhagida). J. Morphol. 274, 258–274. (10.1002/jmor.20089) 23109054

[58] Nebelsick M 1993 Introvert, mouth cone, and nervous system of *Echinoderes capitatus* (Kinorhyncha, Cyclorhagida) and implications of the phylogenetic relationships of Kinorhyncha. Zoomophology 113, 211–232. (10.1007/BF00403313)

[59] Kristensen RM 1991 Loricifera. In Microscopic Anatomy of Invertebrates (eds Harrison FW, Ruppert EE), pp. 351–375. New York, NY: Wiley-Liss.

[60] Montgomery TH 1904 The development and structure of the larva of *Paragordius*. Proc. Natl. Acad. Sci. Phila. 56, 738–755.

[61] Malakhov AA 1994 Nematodes. Structure, development, classification, and phylogeny. Washington, DC: Smithsonian Institution Press.

[62] Mayer G et al. 2013 Selective neuronal staining in tardigrades and onychophorans provides insights into the evolution of segmental ganglia in panarthropods. BMC Evol. Biol. 13, 230 (10.1186/1471-2148-13-230) 24152256 PMC4015553

[63] Loesel R, Wolf H, Kenning M, Harzsch S, Sombke A 2013 Architectural principles and evolution of the arthropod central nervous system. In Arthropod biology and evolution (eds Minelli A, Boxshall G, Fusco G), pp. 299–342. Berlin, Heidelberg: Springer-Verlag.

[64] Kristensen RM, Funch P 2000 Micrognathozoa: a new class with complicated jaws like those of Rotifera and Gnathostomulida. J. Morphol. 246, 1–49. (10.1002/1097-4687) 11015715

[65] Müller MCM, Sterrer W 2004 Musculature and nervous system of *Gnathostomula peregrina* (Gnathostomulida) shown by phalloidin labeling, immunohistochemistry, and cLSM, and their phylogenetic significance. Zoomophology 123, 169–177. (10.1007/s00435-004-0099-2)

[66] Kotikova EA, Raikova OI, Reuter M, Gustafsson MKS 2005 Rotifer nervous system visualized by FMRFamide and 5-HT immunocytochemistry and confocal laser scanning microscopy. Hydrobiologia 546, 239–248. (10.1007/s10750-005-4203-5)

[67] Leasi F, Pennati R, Ricci C 2009 First description of the serotonergic nervous system in a bdelloid rotifer: *Macrotrachela quadricornifera* Milne 1886 (Philodinidae). Zool. Anz. 248, 47–55. (10.1016/j.jcz.2008.10.002)

[68] Orrhage L, Müller MCM 2005 Morphology of the nervous system of Polychaeta (Annelida). Hydrobiologia 535/536, 79–111. (10.1007/s10750-004-4375-4)

[69] Hejnol A, Lowe CJ 2015 Embracing the comparative approach: how robust phylogenies and broader developmental sampling impacts the understanding of nervous system evolution. Phil. Trans. R. Soc. B 370, 20150045 (10.1098/rstb.2015.0045) 26554039 PMC4650123

[70] Arendt D, Nübler-Jung K 1999 Comparison of early nerve cord development in insects and vertebrates. Development 126, 2309–2325. 10225991 10.1242/dev.126.11.2309

[71] Ehlers U, Alrichs W, Lemburg C, Schmidt-Rhaesa A 1996 Phylogenetic systematization of the Nemathelminthes (Aschelminthes). Verh. Dtsch. Zool. Ges. 89.1, 8.

[72] Mayer G, Kauschke S, Rüdiger J, Stevenson PA 2013 Neural markers reveal a one-segmented head in tardigrades (water bears). PLoS ONE 8, e59090 (10.1371/journal.pone.0059090) 23516602 PMC3596308

[73] Schulze C, Neves RC, Schmidt-Rhaesa A 2014 Comparative immunohistochemical investigation on the nervous system of two species of Arthrotardigrada (Heterotardigrada, Tardigrada). Zool. Anz. 253, 225–235. (10.1016/j.jcz.2013.11.001)

[74] Schulze C, Schmidt-Rhaesa A 2013 The architecture of the nervous system of *Echiniscus testudo* (Echiniscoidea, Heterotardigrada). J. Limnol. 72, 44–53. (10.4081/jlimnol.2013.s1.e6)

[75] Strausfeld NJ 2012 Arthropod brains. Evolution, functional elegance, and historical significance. Cambridge, MA: Harvard University Press.

[76] Scholtz G, Edgecombe GD 2006 The evolution of arthropod heads: reconciling morphological, developmental and palaeontological evidence. Dev. Genes Evol. 216, 395–415. (10.1007/s00427-006-0085-4) 16816969

[77] Bailly X, Reichert H, Hartenstein V 2013 The urbilaterian brain revisited: novel insights into old questions from new flatworm clades. Dev. Genes Evol. 223, 149–157. (10.1007/s00427-012-0423-7) 23143292 PMC3873165

